# The State of the Art about Etiopathogenetic Models on Breast Implant Associated–Anaplastic Large Cell Lymphoma (BIA-ALCL): A Narrative Review

**DOI:** 10.3390/jcm10102082

**Published:** 2021-05-12

**Authors:** Roberto Cuomo

**Affiliations:** Plastic and Reconstructive Surgery Unit, Department of Medicine, Surgery and Neuroscience, S. Maria Alle Scotte Hospital, University of Siena, Mario Bracci Street, 53100 Siena, Italy; robertocuomo@outlook.com

**Keywords:** BIA-ALCL, breast implants, texturization, anaplastic large cell lymphoma, breast reconstruction, aesthetic, prosthesis

## Abstract

Background: Breast-implant-associated anaplastic large cell lymphoma is a rare malignancy linked to texturized breast implants. Although many researchers focus on its etiopathogenesis, this topic is affected by a lack of evidence. Materials and Methods: A literature review about BIA-ALCL was made. Results and conclusions: Although the incidence is reported between 1:355–1:30,000, there is great attention to BIA-ALCL. The incidence is uncertain due to many reasons. It may well be lower, due to inclusion in multiple databases as pointed out by the FDA and undiagnosed cases. The role of chronic inflammation, bacterial contamination, and mechanical forces was discussed. Clarification is needed to understand the mechanisms underlying the progression of alterations and mutations for BIA-ALCL; new molecular analysis and pathogenetic models should be investigated.

## 1. Introduction

Breast-implant-associated anaplastic large cell lymphoma is an emerging non-Hodgkin’s lymphoma, often associated with textured-surface implants [1]. It was first described by Keech and Creech in 1997 [2] as a t-cell lymphoma with specific clusters of differentiation. It has been reported as CD 2, 4, 30 and 56 positive and negative for CD 7, 8, TCL1 (tumor cell lysate), EBER (Epstein–Barr encoding region) and ALK (anaplastic lymphoma kinase) [3,4]. Common symptoms are pain, breast swelling, asymmetry, redness, delayed seroma, and solid tumor.

Textured implants were introduced in the 1980s, after manufacturers produced silicone shells with different pore sizes to improve the implant grip into the breast pocket, to reduce movement and, successively, to reduce the incidence of capsular contracture [5,6,7,8].

BIA-ALCL has been linked to textured breast implants [9,10,11,12], with a lifetime risk of 1:30,000 women with textured implants; since 2016, BIA-ALCL has been considered an autonomous entity by the World Health Organization [13]. The prognosis of this cancer is good when detected in an early stage and treated adequately, with risk of death less than 5%, lower than other malignancies [14]. Despite many years of research, the etiopathogenetic mechanisms responsible for it remain poorly understood [1,15,16,17,18,19]. Many researches theorize a role of bacterial biofilm around the implant: this leads to chronic inflammation and, successively, to capsule contracture [3] and/or BIA-ALCL. The link between biofilm, chronic inflammatory response and BIA-ALCL is not the only mechanism proposed: the roles of personal characteristics and mechanical stimulations has been described by many researchers in the world.

The aim and scope of this manuscript is to analyze the state of the art about BIA-ALCL. Data were compared by combining the most recent evidence to discuss the most modern etiopathogenetic theories, and the possible genetic and molecular predisposing factors. Interactions between bacteria and texturing were also highlighted, analyzing the molecular aspects of biofilms on textured surfaces.

## 2. Results

### 2.1. Definition and Epidemiology

BIA-ALCL is a rare T-cell lymphoma, CD30 positive, ALK-negative usually discovered after the diagnosis of spontaneous periprosthetic seroma or intracapsular mass around the breast implant [20].

It belongs to the family of ALCL [13,21]; although its first description dates back to 1997 [2], it received increased attention by the physicians only in 2011, when the United States Food and Drug Administration released a specific communication [22].

BIA-ALCL was initially considered a provisional entity, not distinguishable from ALK-negative anaplastic large cell lymphoma; the first molecular description of BIA-ALCL as an independent type of cancer was obtained in 2019 by Di Napoli et al. [23].

The incidence is very low: in 2019 a total of 573 cases worldwide were registered by the American Society of Plastic Surgery, and the estimated breast-implant surgeries performed were approximately 35 million worldwide [22].

Although the incidence is reported between 1:355–1:30,000 women with textured implants, there is a big bias about the real epidemiology due both a lack of information of implanted devices and about undiagnosed cases [24,25,26,27].

### 2.2. Etiopathogenetic Models

The body starts a complex immunobiological process known as “foreign body reaction” after implantation. It begins with the attempt to phagocytize foreign antigens and continues with the stimulation of chronic inflammation pattern and fibrous capsule formation. This is raised by active neutrophil infiltration and mast cell activity, with temporary matrix; a great number of immune cells migrate on the site of the implant in this first phase. After 10–14 days, the monocytes/macrophages involved to phagocyte the foreign antigens recruit fibroblasts, and extracellular matrix proteins are produced, forming the definitive capsule.

A complete capsule made of collagen fibrils envelops the implant, creating a hypoxic, isolated and immune-privileged space at the end of this process and the foreign body is excluded from the body, but the presence of chronic immune response can remain.

After breast-implant positioning, capsule formation is a tissue response that, in some cases, can deform the device in a process called “capsular contracture” [28]. The implant texturization impacts on this fibrotic tissue, triggering a mechanism of disruption of collagen fibers around the implant: the tissue ingrowth into the texturization disrupting the alignment of fibers, reducing the percentage of risk of capsular contracture. Another characteristic of texturization is the capability to make the implant more adherent to the thoracic wall: deeper textures are linked to major tissue ingrowth, increasing the coefficient of friction and stabilizing the device to the surrounding tissue [6,28,29,30,31,32,33,34,35,36,37,38,39,40].

If texturization improves the implant stabilization on chest wall and reduces the capsular contracture, what happens in BIA-ALCL?

The capsular tissue around the implant is poor in cells’ representation but rich in fibers. The rare cells are inflammatory cells principally, such as lymphocytes and macrophages; in 1996 Katsin et al. showed two important aspects:

- That textured implants involve capsules characterized by a predominant T-cell CD3, CD4, CD29 and CD45RO receptor positive; and
- This kind of implant and capsule were associated with the evidence of silicone-laden macrophages that lead to further T-cell chemiotaxis [41].

Successively, in 2004 and in 2012 Wolfram et al. showed the silicone breast implant ability to stimulate a strong Th1/Th17 response and the presence of T-cells positive for FoxP3 and CD25 [42,43]. These two receptors were identified in the BIA-ALCL population by Di Napoli et al. [23,44]. These data were confirmed by DeCoster et al., suggesting a phenotypic behavior of BIA-ALCL linked to T-regulatory and Th17 cells, and suggesting a role of the capsular macrophages, the silicone and the T-cell response.

The growth of these cells cannot be linked only with the type of implant and/or type of texturization, due to lack of evidence as suggested by many authors [45,46].

Furthermore, many authors underline the role of texturization as an irritative and abrasive mechanism that overstimulates the chronic inflammatory response [22,47], but despite this, the cause (or the trigger) that can transform T-cells’ response in BIA-ALCL is unclear [1].

### 2.3. The Biofilm Role in Breast Implants

Recent evidence suggests a role of antigenic stimulus that overdue a chronic inflammatory state led by some bacteria such as Staphylococcus and Ralstonia pickettii [1,48,49,50,51]. However, in 2013 Deva et al. hypothesized that bacterial contamination that occurred in the time of implant surgery can produce a predominant T-cell response [48,49,52,53,54,55,56,57,58].

Collett et al. analyzed specimens of contracted implant capsules and revealed a predominance of Gram-positive bacterial (*Staphylococcus* spp.); the same author showed that in BIA-ALCL, the predominant contamination was by Gram-negative (principally *Ralstonia pickettii* and *Pseudomonas* spp.). He concluded that Gram-positive bacteria can promote capsular contracture while Gram-negative can lead to lymphocyte simulation and, eventually, transformation [52].

The correlation between infection and capsular contracture is clarified as shown by different researchers [59,60,61,62]. Furthermore, the incidence of capsular contracture is lower when povidone-iodine, gentamicin and cefazolin are used to wash the implant pocket [61,62,63,64,65,66,67].

Hypothesis that underlines the role of biofilm in BIA-ALCL has been proposed by several authors [54] describing models where biofilm produces on high surfaces of implants, leading to chronic antigen stimulation. In patients genetically susceptible, the dysregulation of the JAK1/STAT3 pathway can occur, predisposing the emergence and proliferation of monoclonal CD30+ and ALK-negative cells (although CD30+/ALK-negative cells are not necessarily of malignant nature). Germeline mutations TP53/DNMT3A have been described as DUSP22, and TP63 as well.

Biofilm and texturization are linked by the capability of bacteria to colonize the implant with high area surface, as suggested by many authors [56,68,69].

### 2.4. The Friction Role

The idea about a role of friction forces by the implants on the tissue was proposed by observing data related to other prosthetic materials used by orthopedics [60,70,71,72]. Actually, the cases of anaplastic large cell lymphoma ALK-negative and CD30-positive associated with other kinds of prostheses are very rare, but case reports linked to dental, gluteal, and port device implants and gastric lap bands was published from 2016 to 2019 by Yoon [73], Manikkam Umakanthan [74], Engberg [75] and Shauly [76].

In 2019, Hallab et al. focused on the relationship between the chronic inflammation in orthopedic implants and the chronic inflammation in BIA-ALCL. The debris produced by orthopedic-implant wear leads to macrophages stimulation and phagocytosis, inflammasome activation and secretion of interleukin-family cytokines. The silicone particles can induce a similar pattern of inflammation in animal models, but this results in a fewer responses than metal orthopedic implants, and there is no evidence of an analogue of BIA-ALCL in orthopedic implants [77]. Because of this, other mechanisms should be investigated.

Can these three aspects (texturization, friction and biofilm) be linked to each other?

In 2017 Efanov et al. analyzed double-capsule specimens, proposing a mechanical model of its genesis: the breast implant is always subject to mechanical stress; in macrotextured implants, a detachment from surrounding tissue can happen and, at the same time the macrotexturization determines a fiber disorganization. This produces the formation of a new delaminated capsule [78].

Recently, Calderan et al. analyzed the ultrastructural features of double-capsule connective around macrotextured implants with traditional microscopy, scanning electron microscopy (SEM) and transmission electron microscopy (TEM). They concluded that silicon debris may have a role in the genesis of a double capsule: the debris can alter the disposition of collagen in the capsule, causing delamination. This phenomenon can occur in both micro- and macrotexturization, but the macrotexturization probably involves major adherence between the implant and the capsule, so the movements can create a discontinuity between the connective layers, forming first a space and then a double capsule [79].

However, in 2015 Giot et al. showed an important data series about the colonization of bacteria on a double capsule [80]: the analysis of the specimen revealed that the inner capsule is composed of thin sheets of organized collagen with minimal cell density (mainly fibroblasts and mononuclear cells). Furthermore, they described delamination of the inner capsule. The outer capsule, instead, revealed high cellular density (lymphocytes, monocytes, macrophages) with synovial metaplasia in 70% of cases. The most interesting finding was the bacterial cell density: it was higher on the implant aspect of the inner capsule than in the space between the two capsules.

There is not unanimous consent that connects the single dots of this complex puzzle, and a single theory that unifies the role of biofilm, the friction, and the double capsule, and the BIA-ALCL genesis can be only hypothesized.

### 2.5. The Role of Toll-Like Receptors (TLRs)

Making a comparison with the ophthalmologic lens, we can understand the hypothetic role of TLRs.

The association between inflammatory response and some form of biodeposit in the ophthalmologic contact lens due to bacterial bioburden inside of the internal layer of the lens has been already described. Biodeposits of denatured protein and bacteria can adhere in these spaces, unleashing an immune response. Some researchers demonstrated the involvement of toll-like receptors activated by bacterial protein; they are capable of triggering the innate immune response until adaptive immunity. Toll-like receptor-4 and 2 (TLR-4, TLR-2) recognizes cell wall lipopolysaccharides, respectively of Gram-negative and Gram-positive bacteria [81,82,83,84].

### 2.6. What Happens in Breast Implants?

Bachour et al. in 2019 compared the expression of toll-like receptors in contracted and noncontracted capsules: they found a higher expression of TLR in contracted capsules than uncontracted, and the differences were significant for TLR-2, TLR-6 and TLR-8 [85]. The macrophage activation leads to cytokines and chemokines secretion such as IL-8 and TNF-alpha, producing an increased inflammatory response usually founded in contracted capsules [86,87,88,89,90,91,92,93,94,95,96].

As above exposed, macrotexturization can lead to a double capsule. The internal capsule is thin and strictly adherent; the inner layer of this capsule can lead to a “Velcro effect” [80,90,91] where bacteria can grow and create biofilm. Due to this, the immune system is then stimulated with chronic mechanisms—TLR stimulating themselves because bacteria presence. TLR stimulation also may continue after bacteria are resolved by contact of pathogen-associated molecular pattern clinging to this delaminated and disrupted inner capsule [85,86,96]. This can lead to a long-term stimulation of a Th1 and Th17 response between the two membranes (the inner and the outer layer of double capsule).

Clarification is needed to understand the mechanisms underlying the progression of alterations and mutations of BIA-ALCL; new molecular analysis and pathogenetic models will be needed, and these will consider what above is summarized. In addition to this, particular attention should be paid to the space between the two aspects of the double capsule: the capsule is an isolating barrier between the implant and the organism; the presence of a small space (even virtual) between the outer and the inner layer in the double capsule produces an “atypically isolated environment” with hypothetic lower immune surveillance. In this space, cells can undergo genetic alterations recognized not effectively by the immune system.

## Data Availability

Not applicable.
